# The hidden impact: the rate of nicotine metabolism and kidney health

**DOI:** 10.3389/fendo.2024.1424068

**Published:** 2024-09-17

**Authors:** Xiaona Wang, Shanshan Su

**Affiliations:** Department of Nephrology, Affiliated Hospital of Shandong University of Traditional Chinese Medicine, Jinan, China

**Keywords:** non-linear, nicotine metabolite ratio, kidney function, NHANES, cross-sectional research

## Abstract

**Objectives:**

The effects of nicotine metabolism on the kidneys of healthy individuals have not been determined. The nicotine metabolite ratio (NMR) indicates the rate of nicotine metabolism and is linked to smoking behaviors and responses to tobacco treatments. We conducted this study in order to investigated the relationship between nicotine metabolite ratio (NMR) and kidney function.

**Methods:**

An analysis of cross-sectional data of adults was conducted using a population survey dataset (National Health and Nutrition Examination Survey Data 2013/2018 of the United States). A weighted multivariate regression analysis was conducted to estimate the correlation between NMR and kidney function. Furthermore, we apply fitting smooth curves to make the relationship between NMR and estimated glomerular filtration rate (eGFR) more visualized.

**Results:**

The research included a total of 16153 participants. Weighted multivariate regression analyses adjusted for possible variables showed a negative relationship between NMR and estimated glomerular filtration rate (eGFR).The β (95%CI) of the regression equation between NMR and eGFR was -2.24 (-2.92, -1.55), the trend testing showed consistent results. NMR is positively correlated with urinary albumin creatinine ratio (uACR), but it is not statistically significant. A stratified analysis found a negative correlation between NMR and eGFR in all age, gender and diabetes subgroups, the results were not statistically significant among Mexican Americans and other races. Notably, each unit rise in NMR corresponded to a 4.54 ml/min·1.73m² lower eGFR in diabetic participants and a 6.04 ml/min·1.73m² lower eGFR in those aged 60 and above.

**Conclusions:**

Our study shows that nicotine metabolite ratio is negatively associated with kidney function among most adults. It will be necessary to conduct more well-designed prospective clinical trials in order to determine the exact causal interactions between NMR and kidney function. Specific mechanisms also need to be further explored in basic experiments.

## 1 Introduction

Recent changes in natural and social environments have altered people’s lifestyles. The rise in metabolic diseases highlights the ongoing concern about how human behavior affects physical health. Chronic kidney disease is linked to metabolism, and smoking-induced metabolic changes impacting physiology and pathology are also significant.

Chronic kidney disease (CKD) has become a major public health issue that seriously affects the health of the population. CKD is characterized by declining eGFR and albuminuria (1). There are approximately 10%-15% of people worldwide who suffer from chronic kidney disease, which poses serious public health burden (2). Study confirms that risk factors for CKD include hypertension (3), diabetes mellitus (4), overweight (5), COVID-19 infections (6), nephrotoxic drugs, ageing (7), and air pollution exposure (8).

In 2019, smoking tobacco accounted for 769 million deaths and 200 million disability-adjusted life years, and it was the leading cause of death among men (9). A 2021 survey indicates that approximately 18.7% of American adults currently use tobacco products. Within this demographic, 11.5% use cigarettes, 4.5% use electronic cigarettes, 3.5% use cigars, 2.1% use smokeless tobacco, and 0.9% use pipes (10). Smoking has been identified as a modifiable risk factor contributing to mortality in various diseases, including malignancies, cardiovascular diseases, respiratory diseases, and chronic kidney disease (CKD) (11–13). Research indicates that the link between tobacco use and kidney function is debated. Most studies show smoking harms kidney function, but a few do not find any connection.

Nicotine, the primary agent responsible for smoking addiction, is released and swiftly absorbed in the lungs, subsequently entering the brain where it binds to nicotinic acetylcholine receptors (nAChRs) in neural tissue (14). Nicotine exerts its effects through the activation of nicotinic acetylcholine receptors (nAChRs). Various non-neuronal cell types, including renal cells (such as podocytes, tubular epithelial cells, and mesangial cells), endothelial cells, and smooth muscle cells within the vascular system, are known to express nAChRs (15, 16). Research indicates that nicotine reduces kidney cell viability, induces the production of reactive oxygen species (ROS), and upregulates the expression of the α7 nicotinic acetylcholine receptor (7nAChR) (17). Nicotine exhibits a high affinity for brain tissues in individuals who smoke, thereby enhancing their receptor binding capacity (18). In autopsy samples obtained from human smokers, the kidneys exhibit a pronounced affinity for nicotine (19). Nicotine inhibits the proliferation of HK-2 cells through modulation of the AKT signaling pathway (20). By increasing ROS levels, forming stem cell-like spheres, and altering EMT, chronic nicotine exposure caused HK-2 cells to grow and become cancerous (21).

Nicotine is metabolized in the liver by cytochrome P4502A6, producing cotinine and hydroxycotinine (22). The accuracy of assessing smoking exposure through self-reported smoking habits is limited. Objective measurement of smoking levels can be achieved by quantifying cotinine, a metabolite of nicotine, in biological specimens such as saliva, plasma, and urine. Cotinine and hydroxycotinine are more frequently utilized as biomarkers for active smoking and exposure to tobacco smoke due to their higher potency and extended elimination half-life. The Nicotine Metabolite Ratio (NMR), defined as the ratio of hydroxycotinine (HC) to cotinine (COT), is a significant biomarker in the study of nicotine dependence. A higher NMR is indicative of a more rapid metabolism of nicotine, which is associated with increased levels of nicotine dependence (23–25). As a result, NMR can be used to estimate the risk of tobacco-related diseases. Prior research examining the association between tobacco exposure and renal impairment has predominantly relied on self-reported smoking behaviors. In this study, the nicotine metabolite ratio, utilized as an exposure factor, demonstrated greater accuracy, objectivity, and reliability compared to self-reported smoking data. The nicotine metabolite ratio, indicative of the rate of nicotine metabolism, reflects the degree of nicotine dependence in the human body. This metric may, to some extent, encourage researchers to investigate the intrinsic relationship between smoking and kidney disease at both metabolic and genetic levels.

In this study, we investigated whether there is an association between nicotine metabolite ratio (NMR) and renal function (eGFR and urinary albumin creatinine ratio) among individuals aged 18 and above residing in the United States.

## 2 Materials and methods

### 2.1 Study population

**Figure 1:**
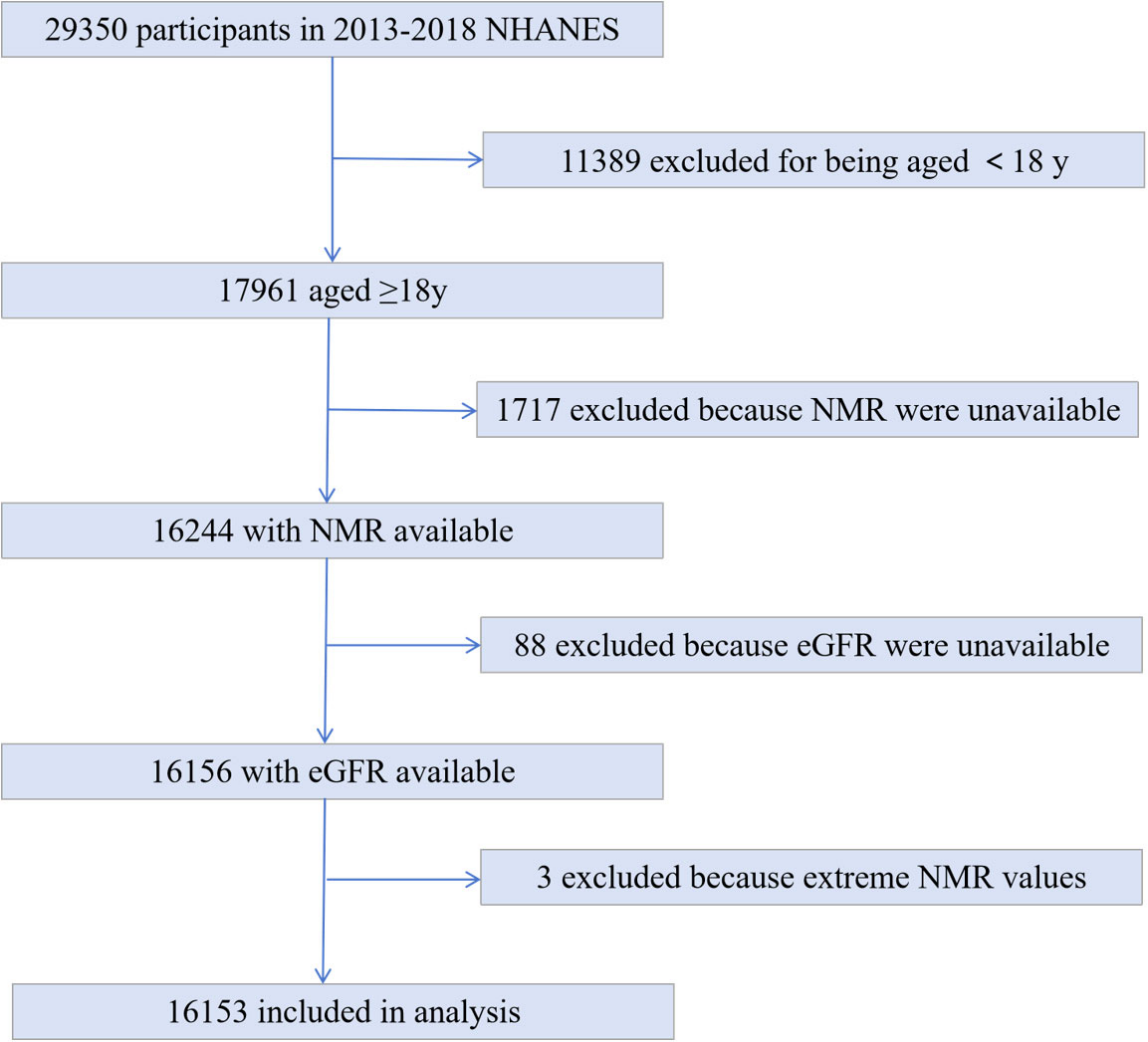
Study flowchart. Flowchart showing study selection. Of 29350 participants in the 2013 to 2018 National Health and Nutrition Examination Survey(NHANES), 16153 remained after fulfilling inclusion and exclusion criteria.

The NHANES database collects health information and nutrition data of the United States population. The database, which began in the 1980s, updates its data every two years in a cycle and is free and open to the public, allowing researchers to download the data directly as needed. The biological samples contain serum, plasma, and urine from the participants and cover a wide range of measures. It also contains data from a large number of questionnaires covering a wide range of demographics, socio-economics, dietary and health concerns, and physical examination including physiological and body measurements, laboratory tests, and more (26). We used NHANES data from 2013-2018 with 29350 participants. 11398 people for being aged < 18y, 1717 with missing hydroxycotinine and cotinine values, 88 participants with serum creatinine unavailable were excluded. 3 excluded because extreme large NMR values. Ultimately, 16153 participants involved in the analyses (**Figure 1**).

### 2.2 Variables

The measurements of serum cotinine and hydroxycotinine are performed using an isotope-dilution high-performance liquid chromatography-atmospheric pressure chemical ionization tandem mass spectrometry (ID HPLC-APCI MS/MS). Additional details of the protocol were previously described (27). The determination of serum creatinine is by enzymatic methods. We calculated eGFR by the CKD-EPI creatinine formula which is based on serum creatinine levels as well as age and race. Research confirms that the CKD-EPI equation outperforms the MDRD equation in the presence of higher GFR, with less bias, more precision and accuracy (28). The following variable were included in our analysis as covariates: age, gender, race, education, BMI, systolic pressure, diastolic pressure, total cholesterol, serum albumin, uric acid, diabetes, CVD, stroke. The CVD was obtained from the medical conditions questionnaire. Those who answered “YES” to the following question “Have you ever been told by a physician or a health professional that you had stroke?” was considered having stroke. The study defined diabetes mellitus as self-diagnosed by a physician, taking oral hypoglycemics, or using insulin supplements, or fasting blood glucose greater than 7.0mmol/L. The detailed information can be found at http://www.cdc.gov/nchs/nhanes/.

### 2.3 Statistical analysis

EmpowerStats (http://www.empowerstats.com) was used for statistical analyses. We conducted a weighted analysis and variance estimation to address the significant variability present in our dataset. We express categorical variables as frequency and percentage distributions (n%). The continuous variables in this study deviate from a normal distribution; therefore, their central tendency and dispersion are represented using the median and interquartile range. For multiple comparisons, the weighted χ² test was used for categorical variables, and the weighted linear regression for continuous variables. A weighted multivariate regression analysis was conducted to estimate the correlation between NMR and kidney function (eGFR and urinary albumin creatinine ratio). The subgroup analysis was succeeded by stratified multivariate regression analyses. To account for the nonlinear relationship between NMR and kidney function, smooth curve fittings were employed. *P* value less than 0.05 was considered statistically significant.

## 3 Results

### 3.1 Characteristic of study population

A total of 16153 participants, aged 18 to 80 years, were included in our analysis. The weighted characteristics of the participants were subclassified according to NMR quartiles, as presented in **Table 1**. Significant differences in baseline characteristics were observed across the NMR quartiles. Participants in the highest NMR quartile, compared to those in other subgroups, were more likely to be female and exhibited higher levels of total cholesterol and educational attainment, as well as lower levels of uric acid and estimated glomerular filtration rate (eGFR). The high NMR group is older with a higher proportion of diabetes patients.

**Table 1:** Baseline characteristics of study participants from 2013 to 2018 National Health and Nutrition Examination Survey.

Nicotine metabolite ratio	Q1 (0.004-0.345)	Q2 (0.345-0.593)	Q3 (0.593-0.998)	Q4 (1.000-5.119)	*P* value
Age (years)	40.00 (27.00,56.00)	46.00 (31.00,61.00)	53.00 (37.00,66.00)	55.00 (38.00,68.00)	<0.0001
Gender, n (%)					<0.0001
Male	2298 (56.91)	2061 (51.05)	1003 (44.11)	2389 (41.16)	
Female	1740 (43.09)	1976 (48.95)	1271 (55.89)	3415 (58.84)	
Race, n (%)					<0.0001
White	1051 (26.03)	1514 (37.50)	983 (43.23)	2409 (41.51)	
Black	1407 (34.84)	918 (22.74)	395 (17.37)	645 (11.11)	
Mexican American	457 (11.32)	509 (12.61)	311 (13.68)	1217 (20.97)	
Other	1123 (27.81)	1096 (27.15)	585 (25.73)	1533 (26.41)	
Education, n (%)					<0.0001
Less than high school	985 (24.39)	947 (23.46)	502 (22.08)	1175 (20.25)	
High school	1098 (27.19)	1056 (26.16)	560 (24.63)	1098 (18.92)	
More than high school	1955 (48.42)	2034 (50.38)	1212 (53.30)	3531 (60.84)	
Body mass index (kg/m^2^)	28.40 (24.30, 33.60)	28.30 (24.30, 33.00)	28.00 (24.30, 32.50)	28.20 (24.40, 32.50)	0.0004
Systolic pressure (mmHg)	122.00 (112.00, 130.00)	124.00 (112.00, 132.00)	124.00 (114.00, 134.00)	124.00 (112.00, 134.00)	<0.0001
Diastolic pressure (mmHg)	70.00 (64.00, 78.00)	70.00 (64.00, 78.00)	70.00 (64.00, 76.00)	70.00 (64.00, 76.00)	0.0001
Albumin (g/dL)	4.20 (4.00, 4.50)	4.20 (4.00, 4.50)	4.20 (3.90, 4.40)	4.20 (4.00, 4.50)	0.0113
Total Cholesterol (mg/dL)	180.00 (157.00, 208.00)	184.00 (159.00, 212.00)	187.00 (160.00, 215.00)	187.00 (161.00, 215.00)	<0.0001
Uric acid (mg/dL)	5.40 (4.50, 6.40)	5.30 (4.40, 6.40)	5.30 (4.40, 6.40)	5.20 (4.30, 6.20)	<0.0001
Diabetic, n (%)					<0.0001
No	3633 (89.97)	3515 (87.07)	1887 (82.98)	4874 (83.98)	
Yes	405 (10.03)	522 (12.93)	387 (17.02)	930 (16.02)	
CVD, n (%)					<0.0001
No	3733 (92.45)	3625 (89.79)	1952 (85.84)	5122 (88.25)	
Yes	305 (7.55)	412 (10.21)	322 (14.16)	682 (11.75)	
STROKE, n (%)					0.0096
No	3910 (96.83)	3894 (96.46)	2168 (95.34)	5570 (95.97)	
Yes	128 (3.17)	143 (3.54)	106 (4.66)	234 (4.03)	
eGFR (ml/min/1.73m^2^)	103.97 (88.13, 118.92)	98.96 (82.04, 113.77)	92.35 (73.69, 108.48)	92.44 (74.72, 108.72)	<0.0001
Urinary albumin creatinine ratio (mg/g)	6.79 (4.44, 13.21)	7.40 (4.86, 13.90)	8.29 (5.20, 17.10)	7.84 (5.06, 16.25)	0.0001

Median (Q1-Q3) for continuous variables: *P*-value was calculated by the weighted linear regression model.

N (%) for categorical variables: *P*-value was calculated by weighted chi-square test. CVD, cardiovascular disease; eGFR, estimated glomerular filtration.

### 3.2 Association between NMR and kidney function

**Figure 2:**
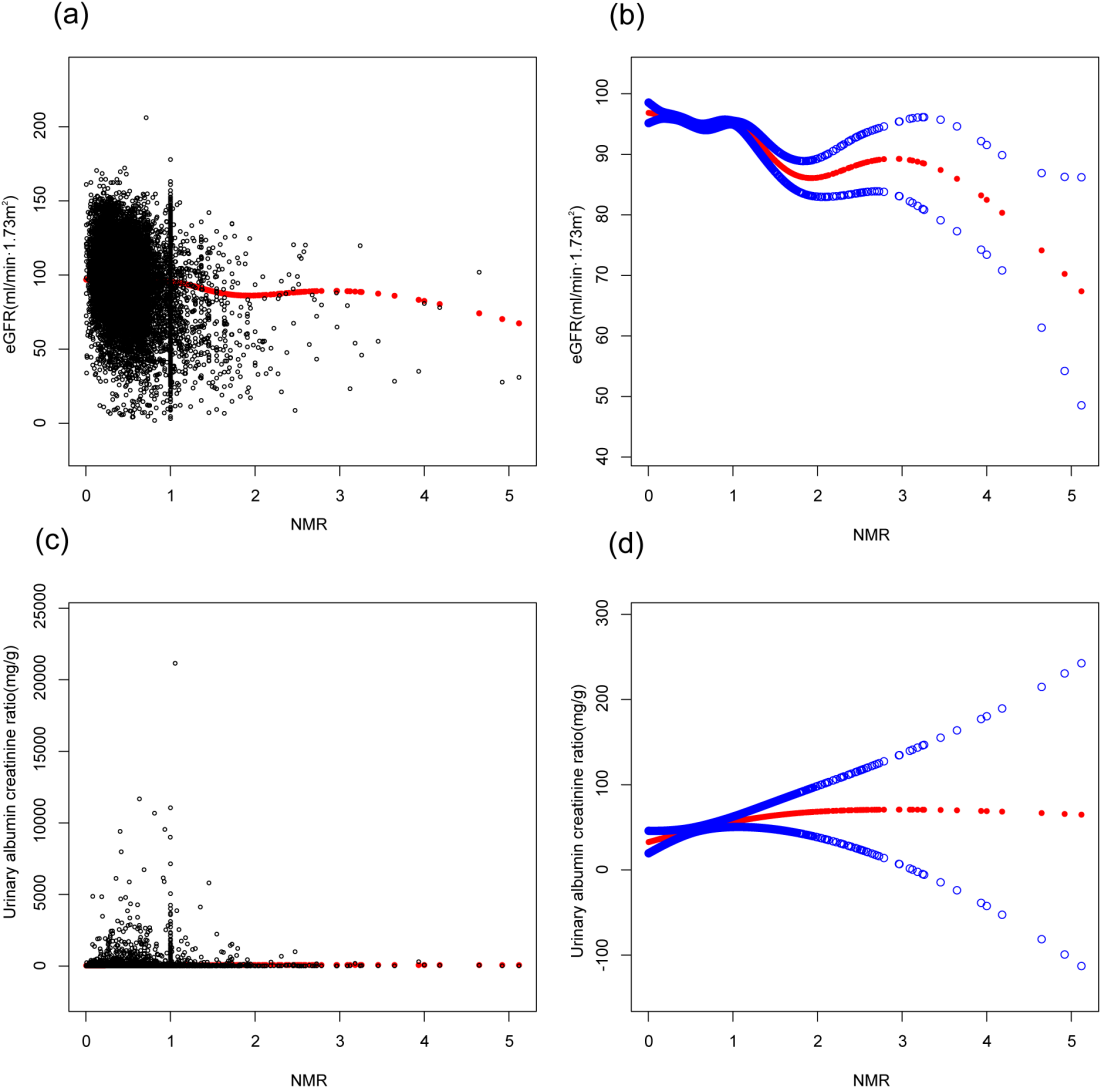
**Figure 2** is based on Model 3. The association between NMR and kidney function. **(A, C)** Each black point represent a sample. The red line represents the curve fitted based on the scatter plot. **(B, D)** Red line represents the smooth curve fit between variables. Blue bands represent the 95% of confidence interval from the fit. Age, gender, race, education, body mass index, systolic pressure, diastolic pressure, total cholesterol, uric acid, serum albumin, diabetes, CVD, stroke were adjusted.

**Figure 3:**
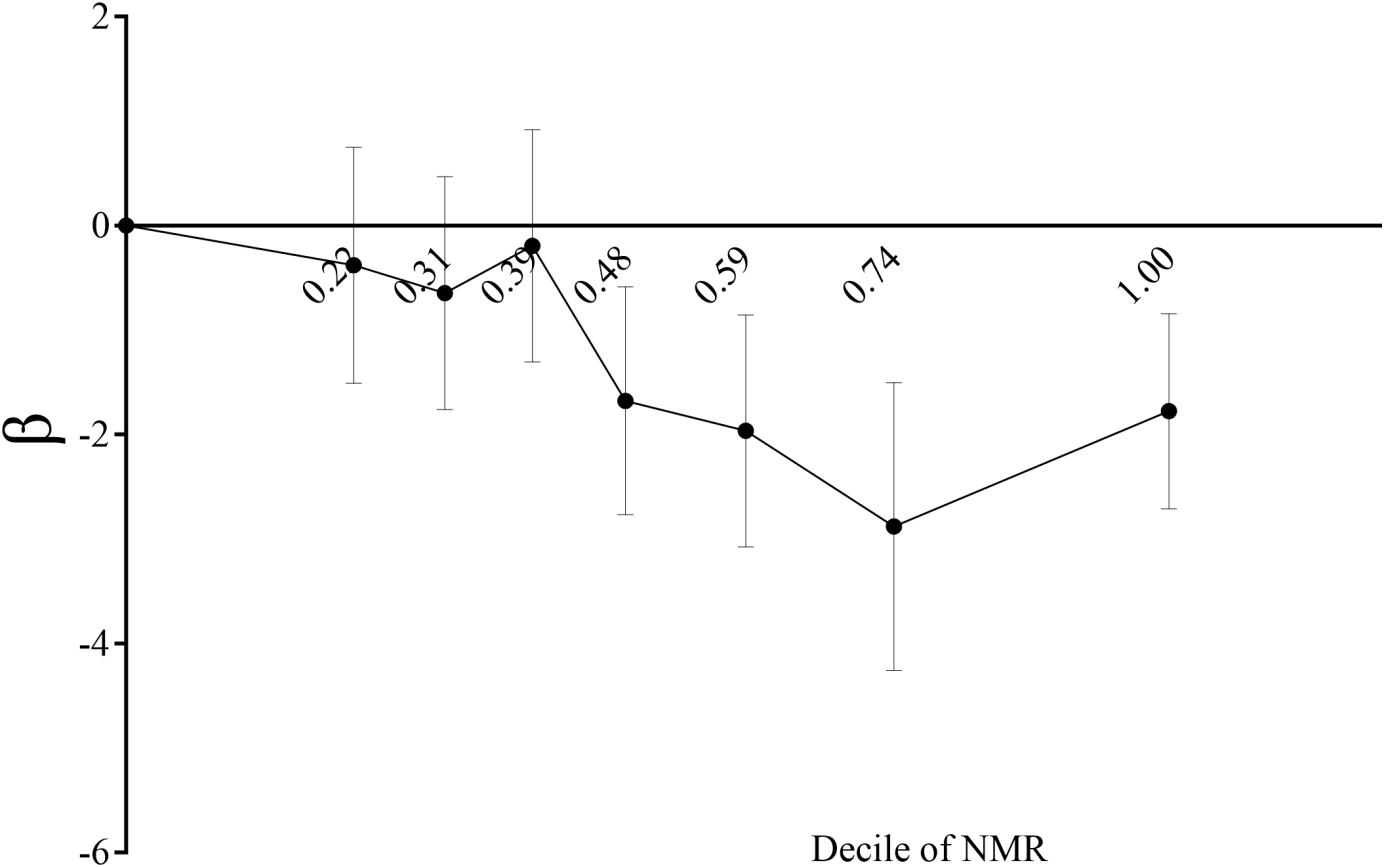
Regression coefficient β of eGFR by decile of serum NMR level plot. Showing regression coefficient β of eGFR by decile of serum NMR level after adjustment for age, gender, race, body mass index, education, systolic pressure, total cholesterol, uric acid, serum albumin, diabetes, CVD, stroke. The vertical lines indicate 95% CIs.

In this study, three models—unadjusted, simple, and fully adjusted—were developed to examine the interaction between nicotine metabolite ratio (NMR) and kidney function, as measured by estimated glomerular filtration rate (eGFR) and the urinary albumin creatinine ratio (uACR) (**Table 2**). Model 2 adjusts for age, gender, and race. Model 3 further includes education, blood pressure, BMI, cholesterol, uric acid, serum albumin, diabetes, CVD, and stroke. In the unadjusted model, a negative association was observed between NMR and eGFR (β = -14.17, 95% CI: -15.13, -13.22, *P* < 0.00001). This inverse relationship persisted in model 2 (β = -2.87, 95% CI: -3.59, -2.16, *P* < 0.00001) and model 3 (β = -2.24, 95% CI: -2.92, -1.55, *P* < 0.00001).We subsequently transformed the NMR data into categorical variables based on quartiles, and trend analysis yielded consistent results. NMR is positively correlated with uACR, but it is not statistically significant. A smooth curve is presented in **Figure 2**, illustrating the relationship between NMR and eGFR after adjusting for all relevant variables. **Figure 3** depicts the regression coefficient β of eGFR across deciles of NMR levels, following comprehensive adjustment for confounding variables.

**Table 2:** The association between NMR and kidney function.

	eGFR			Urinary albumin creatinine ratio		
Exposure	Model 1 *β* (95%CI)	Model 2 *β* (95%CI)	Model 3 *β* (95%CI)	Model 1 *β* (95%CI)	Model 2 *β* (95%CI)	Model 3 *β* (95%CI)
NMR	-14.17 (-15.13, -13.22)	-2.87 (-3.59, -2.16)	-2.24 (-2.92, -1.55)	12.01 (-0.72, 24.75)	7.90 (-5.61, 21.40)	11.82 (-1.52, 25.16)
*P*	<0.00001	<0.00001	<0.00001	0.065	0.252	0.083
Lowest quartile	Reference	Reference	Reference	Reference	Reference	Reference
2nd	-4.49 (-5.51, -3.47)	-0.69 (-1.42, 0.03)	-0.51 (-1.19, 0.18)	8.66 (-4.84, 22.16)	9.28 (-4.34, 22.89)	10.64 (-2.68, 23.96)
3rd	-10.24 (-11.41, -9.06)	-2.40 (-3.24, -1.55)	-1.87 (-2.67, -1.07)	39.51 (23.98, 55.04)	38.02 (22.15, 53.89)	36.09 (20.52, 51.66)
4th	-11.83 (-12.75, -10.90)	-1.66 (-2.35, -0.97)	-1.39 (-2.05, -0.74)	5.31 (-6.94, 17.56)	1.81 (-11.14, 14.76)	8.99 (-3.82, 21.80)
*P* for trend	<0.001	<0.001	<0.001	0.559	0.145	0.888

Model 1: no covariates were adjusted.

Model 2: age, gender, and race were adjusted.

Model 3: age, gender, race, body mass index, education, systolic pressure, diastolic pressure, total cholesterol, uric acid, serum albumin, diabetes, CVD, stroke were adjusted.

### 3.3 Subgroup analysis

**Figure 4:**
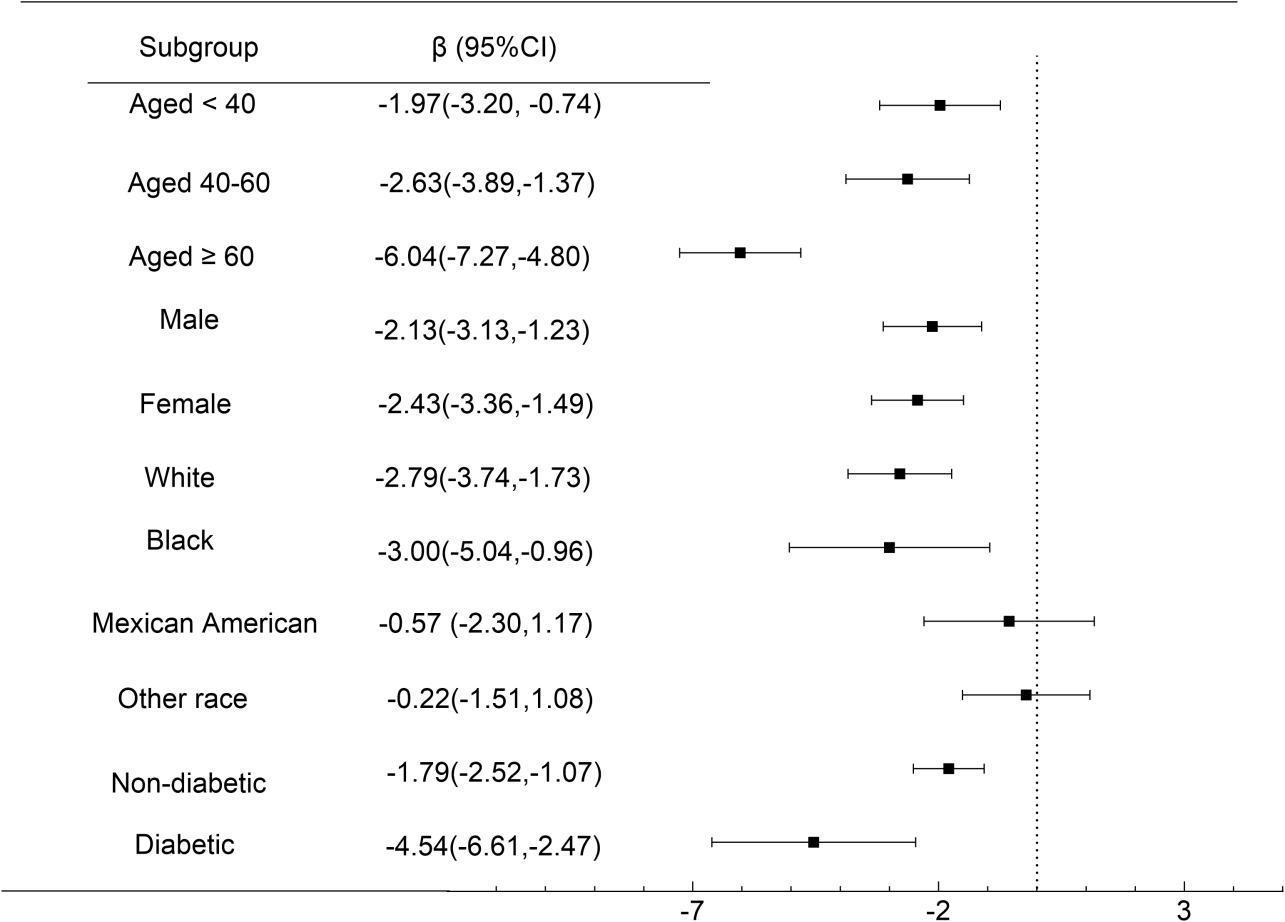
Subgroup Analysis. Forest plot showing the association between NMR and eGFR for various subgroups of participants aged ≥ 18 years in the 2013 to 2018 National Health and Nutrition Examination Survey. Race/ethnicity was self - defined in a written questionnaire. Options were prespecified by the National Health and Nutrition Examination Survey Investigators.

NMR-eGFR relationships stratified by age, gender, race, diabetes are shown in **Figure 4**. The negative correlation of NMR with eGFR remained significant among all age groups, aged < 40 (β = -1.97, 95%CI: -3.20, -0.74, *P* = 0.0017), 40-60 (β = -2.63, 95%CI: -3.89, -1.37, *P* < 0.0001), aged ≥ 60 years (β = -6.04, 95%CI: -7.27, -4.80, *P* < 0.0001), male (β = -2.13, 95%CI: -3.13, -1.23, *P* < 0.0001), female (β = -2.43, 95%CI: -3.36, -1.49, *P* < 0.0001), White people (β = -2.79, 95%CI: -3.74, -1.73, *P* < 0.0001), Black people (β = -3.00, 95%CI: -5.04, -0.96, *P* = 0.0040), non-diabetic(β = -1.79, 95%CI: -2.52, -1.07, *P* < 0.0001), diabetic (β = -4.54, 95%CI: -6.61, -2.47, *P* < 0.0001).It is particularly significant that among participants with diabetes and those aged ≥ 60 years, each unit increase in NMR was associated with a reduction in eGFR by 4.54 ml/min/1.73m² and 6.04 ml/min/1.73m², respectively.

## 4 Discussion

As far as we know, this is the first large-scale research to examine the relationship between nicotine metabolite rate and kidney function. Over 480,000 Americans die each year from smoking. Heart attacks and strokes are two to four times more likely to occur for smokers (29). Smoking increases the risk of atherosclerosis, which leads to narrowing of blood vessels and hypercoagulability, which in turn increases thrombosis (30, 31). Cotinine, hydroxycotinine, and glucuronides account for 85-95% of nicotine excretion in urine (32, 33). According to our findings, NMR is a risk factor for renal impairment. Measurement of NMR levels may therefore be involved in screening models for chronic kidney disease as well as prognostic models to guide therapeutic interventions.

It is biologically plausible that tobacco exposure might interact with kidney function. Cigarette smoking can elevate carboxyhemoglobin levels, platelet activation, and prothrombotic factors (34). As a result, the kidneys experience inflammation, oxidative stress, and endothelial cell dysfunction (35, 36). In addition, smoking induces proteinuria and kidney function abnormalities through advanced glycation end - products, which increases vascular permeability (37). The principal mechanisms underlying the interaction between smoking and the onset and progression of chronic kidney disease in adults include glomerulosclerosis, oxidative stress, endothelial damage leading to mesangial proliferation, and tubulointerstitial fibrosis (36, 38, 39). Research showed nicotine promoted podocyte apoptosis by activating MAPK kinases and enhancing oxidative stress (40). According to recent studies, nicotine stimulation has been shown to induce apoptosis or epithelial-mesenchymal transition in renal tubular epithelial cells through the upregulation of reactive oxygen species (ROS) production (41). In mesangial cells, nicotine activates TGF- or Wnt/-catenin pathways, promoting cell growth and production of extracellular matrix (42, 43). A recent study has demonstrated that nicotine exerts deleterious effects on renal function by modulating the 7nAChR, NLRP6 inflammasome, endoplasmic reticulum (ER) stress, and autophagy pathways (17).

Slow nicotine metabolizers exhibit lower levels of inflammation (ICAM-1 and PGEM) compared to fast metabolizers, which is linked to kidney damage (44). Various cell types exhibit responses to ICAM-1, which serves as a marker for inflammation and chronic disease. (45). NMR serves as an indicator for both exposure to smoke and the biological impact of tobacco. A comprehensive review revealed that individuals who metabolize nicotine at a normal or rapid rate tend to smoke more cigarettes per day compared to those who metabolize it slowly (46). Having a higher NMR indicates greater exposure to kidney-damaging substances from smoking, such as volatile organic compounds and polycyclic aromatic hydrocarbons. Considering that the ratio of nicotine metabolites represent CYP2A6 activity, kidney injury may induce CYP2A6 enzyme activity, leading to an increase in nicotine metabolism rate. Limited knowledge exists regarding the impact of renal pathological changes on other pharmacokinetic parameters in relation to the NMR. Consequently, caution is advised when interpreting kidney-induced increases in the NMR as direct indicators of enhanced CYP2A6 activity.

In our study, a negative relationship was found between NMR and kidney function of 16153 survey participants. Our research results regarding glomerular filtration rate are consistent with previous studies. With increasing NMR, eGFR levels declined significantly. However, our study did not find a correlation between NMR and urinary albumin creatinine ratio. CKD incidence rate was significantly higher for smokers (47, 48). A prospective cohort study identified a vertical relationship between tobacco exposure and albuminuria, independent of diabetes (49). Further, prospective studies have shown that smoking contributes to impairment of kidney function in idiopathic membranous nephropathy (50) and IgA nephropathy (51, 52). In general healthy populations, the effect of smoking on kidney function is unclear. Smoking is associated with CKD, characterized by proteinuria or declining glomerular filtration rate, according to a study published in 2000 (53). A study showed the eGFR changes in smoking subjects were significantly smaller than those in non-smoking subjects (54). The risk of albuminuria was higher in smokers in a cross-sectional study (55). Among patients with stage III-V CKD, a 50% reduction in eGFR or the development of ESKD were not independently associated with current and former smokers (56). It has also been shown that smoking status does not correlate with the presence of proteinuria (57).

As recommended by the STROBE statement, we conducted further subgroup analyses to preferably represent the data set (58). Our findings indicated that a higher NMR was associated with a lower eGFR in both men and women. A study showed end-stage renal disease development was associated with smoking status in men, not in women (59). In addition, according to the subgroup analysis of racial stratification, NMR and eGFR are negatively correlated in White people and Black people, and the relationship is statistically significant, but not in Mexican American and other race. Race-specific differences may be explained by genetic risk factors, obesity status, and other factors. The association was more pronounced among older and diabetic participants. The results of this study were in accordance with previous studies suggesting smoking cigarettes was a significant risk factor for CKD in diabetic patients. A study from South Korea revealed that persistent smokers were 2.17 times far more likely to develop diabetic kidney disease than non-smokers (60). A similar conclusion was drawn in a study of people with type 1 diabetes, which estimated the 12-year cumulative risk of microalbuminuria, macroalbuminuria, and ESRD in current smokers, former smokers, and non-smokers (61).

Since we employed a national sample, our research can to some extent represent the population. The limitation of our study cannot be ignored. As a cross-sectional design, our research limits the inference of a causal relationship between NMR and kidney function. It is therefore necessary to conduct basic pharmacological studies and large-scale prospective studies in order to determine exactly how NMR influences kidney function. Furthermore, As a secondary analysis, we were not able to collect new data, so unmeasured covariates could potentially lead to residual confounding. Due to the fact that this study utilized a database that only consisted of patients from the United States, caution should be exercised when generalizing the findings to populations from other ethnicities. In view of the significant effect NMR has on renal function in elderly individuals and patients with diabetes, it may be possible to reduce the burden on these populations by regulating smoking within an appropriate range. However, the limitations of our research design preclude further elucidation of this matter.

## 5 Conclusions

According to our study, a negative correlation between NMR and kidney function was found. The correlation is particularly pronounced in individuals over the age of 60 and within the diabetic subgroup. Thus, Nicotine Metabolite Ratio (NMR), which indicates an individual’s rate of nicotine metabolism and is known to be detrimental to various physiological systems, may also impair renal function in adults. Smoking must be addressed in order to improve kidney health and reduce the risk of related health problems, particularly for elderly and diabetic patients. As part of chronic kidney disease management, healthcare providers should assess smoking status and provide support for quitting or reducing smoking. In addition, further research is needed on the pathological effects of nicotine or nicotine metabolism on the kidneys.

## Data Availability

The survey data are publicly available on the Internet for data users and researchers throughout the world: https://www.cdc.gov/nchs/nhanes/index.htm.
